# Factors related to retention in a longitudinal study of infants at familial risk for autism

**DOI:** 10.1002/jcv2.12140

**Published:** 2023-01-26

**Authors:** Sally Ozonoff, Monique M. Hill, Alesha Hill, Kevin Ashley, Gregory S. Young

**Affiliations:** ^1^ Department of Psychiatry and Behavioral Sciences University of California Davis Sacramento California USA

**Keywords:** attrition, autism, external validity, internal validity, longitudinal study, retention

## Abstract

**Background:**

Reporting retention data is critical to determining the soundness of a study's conclusions (internal validity) and broader generalizability (external validity). Although selective attrition can lead to overestimates of effects, biased conclusions, or overly expansive generalizations, retention rates are not reported in many longitudinal studies.

**Methods:**

We examined multiple child‐ and family‐level factors potentially associated with retention in a longitudinal study of younger siblings of children with autism spectrum disorder (ASD; *n* = 304) or typical development (*n* = 163). The sample was followed from the first year of life to 36 months of age, for up to 7 visits.

**Results:**

Of the 467 infant siblings who were consented and participated in at least one research visit, 397 (85.0%) were retained to study completion at 36 months. Retention rates did not differ by familial risk group (ASD‐risk vs. Low‐risk), sex, race, ethnicity, age at enrollment, number of children in the family, maternal employment, marital status, or parent concerns about the child at enrollment. A stepwise regression model identified 4 variables that, together, provided the most parsimonious predictive model of study retention: maternal education, maternal age at child's birth, travel distance to the study site, and diagnostic outcome classification at the final study visit.

**Conclusions:**

The retained and not‐retained groups did not differ on most demographic and clinical variables, suggesting few threats to internal and external validity. The significantly higher rate of retention of children diagnosed with ASD (95%) than typically developing children (83%) may, however, present biases when studying recurrence risk. We conclude by describing engagement and tracking methods that can be used to maximize retention in longitudinal studies of children at risk of ASD.

**Abbreviations**
ADOS‐2: Autism diagnostic observation schedule second edition
ASD: Autism spectrum disorder
MSEL: Mullen scales of early learning
Non‐TD: Non‐typically developing
TD: Typically developing

**Key points**

- Selective retention in longitudinal studies can threaten the soundness of their conclusions (internal validity) and their generalizability to broader populations (external validity).
- Few longitudinal studies of children developing ASD report retention rates. If selective attrition occurs, this could skew the results of studies that examine recurrence risk, diagnostic stability, and symptom severity.
- The current study found only four factors that predicted retention, most of which did not present major threats to internal or external validity. However, the significantly higher retention rate of participants who developed ASD compared to those found to be typically developing could bias results, particularly in studies examining recurrence risk.
- Publications of longitudinal cohorts should report both rates of retention and analyses comparing retained and not‐retained samples to examine the effects of attrition on variables important to study validity and generalizability.

Longitudinal research designs are commonly employed to study phenomena that change over time, such as the emergence of typical and atypical behaviors during early development. In the past 2 decades, prospective study designs have been widely used to chart the onset and early course of autism spectrum disorder (ASD). Retention of study participants over the course of data collection, from enrollment shortly after birth through the window when a diagnosis can reliably be made, is critical. In studies of infants at risk for ASD due to a positive family history, some data collected earlier in infancy is not interpretable until diagnostic outcomes have been documented. Participants who do not complete the study may be missing this critical information, rendering much of their earlier collected data unusable. Attrition in longitudinal studies also reduces statistical power and inflates research costs.

It has been recognized for many years that attrition may also present threats to causal inference by impacting the internal and external validity of longitudinal studies in ways that are less relevant to cross‐sectional studies (Campbell & Stanley, 1963). Internal validity refers to confidence that findings are sound and not influenced or confounded by other factors or variables. Internal validity is largely determined by study design and procedures—blinding, randomization, selection of measures—but can also be influenced by attrition if those who do not complete the study share something in common that could provide an alternative explanation for findings or bias the conclusions. External validity refers to the extent to which results from a study can be applied to other samples, situations, or settings. This is driven initially by selection criteria, but attrition can produce biases if those who remain in the study are not representative of the initial sample or the population to which it is hoped to generalize.

Despite the importance of participant retention, many longitudinal studies do not report retention rates or examine effects of attrition on the data. A systematic review of 60 longitudinal studies of health education (Barry, 2005) found that 48% did not record retention rate, and of those that did, less than half examined whether those dropping out differed in systematic ways from those analyzed. Similarly low rates of reporting attrition were found in a review of 57 publications of very preterm infants (Teixeira et al., 2021). These authors suggested that journals should require publications of longitudinal cohorts to report attrition data.

Although retention data are often not well described, when they are reported, several determinants of retention are commonly identified in longitudinal samples. These include parental age, education level, minority status, and distance from the study site. Factors associated with retention are largely similar across different types of samples, including typically developing adolescents (Ewing et al., 2022), older adults (Heid et al., 2021), and young children with ASD (Bradley et al., 2018).

Studies of infants at elevated likelihood of developing ASD often report rates of missing data or missed visits and may account for it through imputation and other statistical methods (e.g., Bussu et al., 2018; Landa et al., 2013; Ozonoff et al., 2015); however, such studies have not reported retention rates. The threat to internal validity of systematic attrition in infant sibling studies is particularly relevant when the focus is on diagnostic rates, timing of symptom emergence, diagnostic stability, and longitudinal follow‐up into later childhood, since biases may be introduced by selective loss of participants with specific characteristics. As one example, estimates of recurrence risk could be inflated if retention rates are higher in children with developmental concerns or signs of ASD than in children who appear to be developing typically. The one published study focused on the risk of ASD recurrence in families with an affected child and a new infant did not report retention data (Ozonoff et al., 2011).

Thus, studying retention is critical in a number of ways, from helping researchers develop strategies to increase engagement and address barriers to completing a longitudinal study, to increasing the likelihood that conclusions will be sound and broadly generalizable. In the current paper, we report on family‐level demographic and child‐level clinical variables predictive of retention in a 3‐year longitudinal study of infants at higher or lower risk of ASD due to family history.

## METHOD

### Participants

The study was conducted in Sacramento, California at the UC Davis MIND Institute. Younger siblings of children with autism spectrum disorder (ASD‐risk group) or typical development (Low‐risk group) were recruited, primarily from the greater Sacramento Valley (median distance from study site = 25.5 miles). Some families who lived further away were also enrolled if they committed to travel to the MIND Institute for assessments. Inclusion criteria for the ASD‐risk group were status as a younger sibling of a child with ASD, with the older sibling's diagnosis confirmed by meeting ASD criteria on both the Autism Diagnostic Observation Schedule (ADOS‐2; Lord et al., 2012) and the Social Communication Questionnaire (Berument et al., 1999). Exclusion criteria for the ASD‐risk group included birth before 34 weeks of gestation and a known genetic disorder (e.g., Fragile X syndrome) in the older affected sibling. The primary inclusion criterion for the Low‐risk group was status as a younger sibling of a child (or children) with typical development, confirmed by an intake screening questionnaire and scores below the ASD range on the Social Communication Questionnaire. Exclusion criteria for the Low‐risk group were participant birth before 37 weeks of gestation, developmental or learning disabilities in any older sibling, and ASD in first‐, second‐, or third‐degree relatives.

Recruitment of infants occurred between 2003 and 2018 in three cohorts corresponding with three funding periods. Recruitment occurred from the prenatal period through 18 months of age (*M* = 4.1 months, *SD* = 5.7 months), with most participants (87%) recruited by 9 months of age, less than 5% recruited after 12 months, and only 1.3% recruited at 18 months. Recruitment methods included community outreach events, the MIND Institute clinic, other MIND Institute study referrals, UC Davis Health billing records, and word of mouth. The first point of contact was initiated by parents who called the study phone number listed in recruitment materials to express interest. Study personnel administered an intake screening interview via telephone to prospective families to determine eligibility. The interview collected date of birth and gestational age, qualifying diagnostic status of an older sibling, exclusionary family history information, referral source, and contact information. Those who met eligibility criteria were either scheduled for their first visit at the end of the intake interview or informed that they would receive a follow‐up contact for scheduling closer to the date of the first visit, depending on the current age of the infant. Parental informed consent was obtained at the first visit. All procedures were approved by the University of California Davis Institutional Review Board.

The intake interview was administered to the parents of 816 infants to determine study eligibility. Of these, 165 infants were determined to be ineligible during intake interviews (e.g., had a known, exclusionary genetic disorder, infant was out of age range, gestational age was below 34 weeks, etc.), and 50 parents decided not to participate after learning more about the study requirements. No data was collected on these 215 infants. This left 601 eligible infants, 396 of whom had one or more older siblings with ASD and 205 who had older sibling(s) who were typically developing. Thirty‐nine of these infant siblings were recruited from a family who had previously enrolled another infant sibling; therefore, the sample of 601 infant siblings derived from 562 separate families. For purposes of analysis, only the most recently enrolled child from a given family was included as the indicator of study retention at the family level. There was no significant difference in retention rates for families with more than one enrolled child (86.1%, CI = 70.7%–94.1%) versus families with only one child enrolled (83.8%, CI = 0.80%–0.87%; *p* = .71).

Of the 562 families eligible to participate, 95 decided not to enroll; the only data collected on these families was recruitment group (ASD‐risk vs. Low‐risk) and referral source. A total of 467 families (304 ASD‐risk and 163 Low‐risk) were enrolled, defined as giving informed consent and completing at least one study visit. Informed consent was obtained at the first visit, documented by the signature of one parent on an IRB‐approved consent form. Visits were conducted longitudinally at up to 7 ages (6, 9, 12, 15, 18, 24, and 36 months). Each visit took approximately 2–3 h to complete, depending on the age of the child.

For the primary analyses, a retained family was defined as an enrolled family (i.e., one who had signed a consent form and completed at least one visit) who also completed the 36‐month visit. A not‐retained family was defined as an enrolled family who did not have a 36‐month visit.

### Retention procedures

#### Participant tracking database

A custom database was created to track participant information. Tracking variables included the reason (e.g., intake, scheduling, sending report) and mode (e.g., email, phone call) of each contact, visit completion status, developmental concerns identified at each visit, diagnostic status, current age of the child, scheduling window for the next visit, and other pertinent information (e.g., preferred mode of contact, best contact times, etc.).

#### Scheduling

At the completion of each visit, families were reminded of when the next visit was due and told that they could request an interim appointment if any developmental concerns arose before then. Weekly scheduling attempts were made using the family's preferred mode of contact (email, text, or phone call) beginning 6 weeks ahead of each visit target date and continuing through the scheduling window (4–6 weeks post‐target date). When a family did not respond to scheduling attempts in the first 2 weeks, the contact method was varied (e.g., work phone, other parent email) and the day of the week and time of day that contacts were attempted was broadened. In cases in which phone and email were no longer in service, contact information for an extended family member that was collected at the first visit was utilized. In instances where the family was still unreachable, further attempts to contact the family were made by utilizing public records tracking services to find current information.

#### Clinical feedback and reports

Families were given verbal feedback about their child's development after every visit and provided with written reports beginning at the 18‐month visit and at all subsequent ages. Verbal feedback and written reports included behavioral observations, scores on normed measures, interpretation of the child's developmental status, including any concerns noted, a reminder of the age of the next visit, and a statement of gratitude for the family's continued participation. When relevant, a diagnostic formulation section, including *DSM* criteria met and formal diagnoses, was also included, as well as treatment recommendations, referrals, and community resource information.

#### Compensation

Families received financial compensation for attending each study visit and additional compensation for completing questionnaires before or at each visit. Families were also offered reimbursement for travel expenses on request.

#### Study “graduation” certificate

Following the completion of the 36‐month visit, families were given a certificate of participation including their child's name and photos of them taken at previous study visits, beginning at their first appointment and ending with the most recent visit. The purpose of the certificate was to provide a token of appreciation and a visual reminder of the longitudinal relationship the family had established with the study and the significance of their contribution over time.

### Measures

#### Demographics questionnaire

Basic demographic information, including parental age, education, employment, and marital status, family income, race, ethnicity, and sibling information was collected at the first visit and, for retained participants, updated at the 36‐month visit. Mailing address was confirmed prior to each visit since questionnaires were sent in advance via U.S. postal service; therefore, travel distance was up to date at the exit visit for both retained and not‐retained participants. See Table 1.

**TABLE 1:** Descriptive characteristics of the retained and not‐retained groups

Variable	Retained	Not retained
Recruitment group, *n* (%)		
ASD‐risk	255 (64.2)	49 (70)
Low‐risk	142 (35.8)	21 (30)
Sex, *n* (%)		
Male	226 (56.9)	36 (48.6)
Female	171 (43.1)	34 (51.4)
Race, *n* (%)		
African‐American	11 (2.8)	2 (2.9)
American Indian	3 (0.8)	0
Asian‐American or Pacific Islander	39 (9.8)	5 (7.1)
White	267 (67.3)	46 (65.7)
More than one race	70 (17.6)	7 (10.0)
Not reported	7 (1.8)	10 (14.3)
Ethnicity, *n* (%)		
Hispanic/Latinx	70 (17.6)	20 (28.6)
Not Hispanic/Latinx	310 (78.1)	41 (58.6)
Not reported	17 (4.3)	9 (12.8)
Cohort, *n* (%)		
1	135 (34.0)	26 (37.1)
2	141 (35.5)	27 (38.6)
3	121 (30.5)	17 (24.3)
Child age at enrollment in months (*M*, SD)	7.36 (3.82)	6.90 (3.35)
Maternal age at child's birth in years (*M*, SD)	33.85 (4.75)	31.02 (4.89)
Number of children (*M*, SD)	2.65 (1.03)	2.49 (1.02)
Household income (%)		
$100k or greater	170 (42.8)	18 (25.7)
$50k to $100k	111 (28.0)	17 (24.3)
$50k or less	77 (19.4)	22 (31.4)
Not reported	39 (9.8)	13 (18.6)
Maternal education (%)		
Graduate degree	103 (25.9)	9 (12.9)
College degree	147 (37.0)	23 (32.9)
Some college	96 (24.2)	14 (20.0)
High school or less	23 (5.8)	10 (14.3)
Not reported	28 (7.1)	14 (20.0)
Maternal employment (%)		
Full‐time	112 (28.2)	13 (18.6)
Part‐time	81 (20.4)	11 (15.7)
Not working	151 (38.0)	34 (48.6)
Not reported	53 (13.4)	12 (17.1)
Marital status (%)		
Married	372 (93.7)	57 (81.4)
Single	22 (5.5)	6 (6.6)
Not reported	3 (0.8)	9 (12.9)
Travel distance (*M*, SD)	63.71 (197.72)	258.77 (536.01)
# General concerns at intake (*M*, SD)	0.13 (0.34)	0.11 (0.32)
# ASD concerns at intake (*M*, SD)	0.13 (0.34)	0.16 (0.37)
Outcome classification at exit (%)		
ASD	52 (13.1)	3 (4.3)
Non‐ TD	97 (24.4)	11 (15.7)
TD	248 (62.5)	52 (74.3)
Missing	0	4 (5.7)
Proportion rescheduled visits (*M*, SD)^a^	0.22 (0.26)	0.24 (0.51)
Contacts re: enrollment (*M*, SD)	4.75 (3.61)	3.61 (3.56)
Contacts re: scheduling per visit (*M*, SD)	2.95 (1.86)	1.10 (1.25)

^a^ Calculated as a proportion, since number of expected visits differed by cohort (up to 7 visits for cohort 2, up to 5 visits for cohorts 1 and 3) and, for not‐retained participants, timing of last visit.

#### Parent concerns

Using a measure previously developed in our laboratory (Ozonoff et al., 2009), parent concerns were collected during the intake interview in response to the prompt: “Do you have any current concerns about your child's development or behavior?”. Responses were coded by the intake interviewer into eight categories of concerns, which were then collapsed into two variables for analysis: ASD concerns (the sum of speech/language, social, stereotyped/repetitive behavior, and unspecified autism concerns), and general concerns (the sum of concerns falling into motor, medical/regulatory, behavior/temperament, and general development categories). Intake interviewers were trained in coding parent concerns to 80% reliability with a training set.

#### Mullen Scales of Early Learning (MSEL; Mullen, 1995)

This standardized assessment of development for children birth to 68 months was administered at all study visits to assess developmental progress and determine outcome classification. Subscales include Gross Motor, Visual Reception, Fine Motor, Receptive Language, and Expressive Language and T scores (mean = 50, SD = 10) are derived based on age norms.

#### Autism diagnostic observation schedule, second edition (ADOS‐2; Lord et al., 2012)

This semi‐structured play‐based interaction and observation was administered to all participants at the 18‐, 24‐, and 36‐month visits to assess symptoms of ASD and determine outcome classification. The ADOS‐2 was also administered to confirm the diagnostic status of an older sibling for the purpose of recruitment group assignment when diagnostic reports including an ADOS score were unavailable for the proband.

#### Outcome classification at exit

Participants were classified into one of three mutually exclusive outcome groups (ASD, Typically Developing, or Non‐Typically Developing) based on data collected at their most recently competed visit. For retained participants, this was done based on 36‐month data, using scores on both the MSEL and the ADOS‐2. The ASD classification was defined as a comparison score of 4 or above on the ADOS‐2 and meeting *DSM* criteria for ASD, verified by a licensed clinical psychologist. The Typically Developing (TD) outcome definition required MSEL T‐scores of 30 or above on all subscales and 35 or above on at least two subscales, as well as an ADOS comparison score of 1 or 2. The Non‐Typically Developing (Non‐TD) group included participants who did not meet *DSM* criteria for ASD and had either an ADOS‐2 comparison score of 3 or greater or MSEL T‐scores below 30 on any subtest or below 35 on two subtests.

Outcome classification for not‐retained participants was based on the last visit completed. If the final visit was at 18 or 24 months, the same criteria were used as for retained participants. However, if the final visit was between 6 and 15 months of age, before the ADOS‐2 was administered in this study, outcome classifications were determined using *DSM‐5* criteria, examiner clinical concerns, and scores on the MSEL. At these ages, the ASD classification was defined as meeting *DSM‐5* criteria for ASD, verified by a licensed clinical psychologist, but none of the not‐retained participants whose final visit was prior to 18 months met these criteria. The TD outcome definition required MSEL T‐scores of 30 or above on all subscales and 35 or above on at least two subscales. Most participants (*n* = 33; 82.5%) in the not‐retained group who exited the study before 18 months were classified as TD. The Non‐TD group included seven participants who did not meet *DSM* criteria for ASD but had: (1) examiner concerns that ASD might be emerging in the future (e.g., child did not meet criteria at the current visit but the examiner felt that he/she might meet criteria at a later visit) and/or (2) MSEL T‐scores below 30 on any subtest or below 35 on two subtests.

This procedure resulted collectively, across the retained and not‐retained groups, in the following outcome classifications: ASD (*n* = 55; 53 ASD‐risk; 11 females); Non‐TD (*n* = 108; 84 ASD‐risk, 42 females); TD (*n* = 300; 165 ASD‐risk, 151 females). Four participants, all in the not‐retained group, did not have enough data to allow classification (2 ASD‐risk, 1 female).

### Analytic plan

The analysis began by examining differences between families who did and did not enroll in the study and whether enrollment refusal rates varied as a function of recruitment group or referral source. The rest of the analyses focused on the 467 enrolled families (i.e., consented and completed at least one study visit), comparing those who were retained (completed the study with a 36‐month visit) to those who were not retained (ended participation in the study prior to the 36‐month visit). Using simple comparisons (chi‐square analyses or *t*‐tests) we examined whether retention rates (retained = 1 vs. not retained = 0) varied as a function of multiple demographic variables, including recruitment group, household income, racial or ethnic minority status, maternal education, and others. In order to identify relative predictive utility of the same set of variables, we utilized a stepwise multiple logistic regression model to identify factors that were each uniquely predictive of study retention and examined the overall predictive utility of the resultant set of factors. Unique predictive value of each variable was assessed by comparing −2Log‐likelihood values between models with and without each variable and testing the difference as a chi‐square distribution. All analyses were conducted in R, version 3.6.1 (R Core Team, 2019).

## RESULTS

### Enrollment refusal

Of the 562 eligible families, 95 (16.9%) decided not to participate after initial contact. There was no difference in the rate of enrollment refusal by recruitment group: ASD‐risk 17.4%, CI = 13.9%–21.6% vs. Low‐risk 15.5%, CI = 11.0%–21.3%; Chi‐square = 0.34, df = 1, *p* = .56. For the 529 families with referral information, there was no significant effect on the probability of enrollment refusal by referral source (Chi‐square = 3.76, df = 3, *p* = .29). Mailings had the highest rate of refusal (18.2%, CI = 10.6%–29.3%), followed by recruitment from other MIND Institute studies and clinics (15.2%, CI = 11.5%–19.9%), public outreach (14.3%, CI = 8.80%–22.4%), and word of mouth (8.00%, CI = 3.6%–16.7%). There was no interaction between recruitment group and referral source on rates of enrollment refusal (Chi‐square = 5.52, df = 3, *p* = .14).

### Study retention

Of the 467 infant siblings who were consented and participated in at least one research visit, 397 (85.0%) were retained to study completion at 36 months. Of the 70 (15.0%) who were not retained to 36 months, 9 (12.9%) had their final visit at 6 months, 32 (45.7%) had their final visit at 12 months, 11 (15.7%) had their final visit at 18 months, and 18 (24.7%) had their final visit at 24 months. The not‐retained families completed an average of 2.50 (SD = 1.40) research visits before dropping out, significantly less than the retained families (*t* = 17.25, *p* < .001) who completed an average of 5.39 (SD = 1.27) research visits. Reasons for dropping out of the study were: moved out of area (31.4%), too busy/unable to attend (31.4%), lost to follow‐up contact (30.0%), and travel/transportation issues (7.1%). There were no differences in reasons given for dropping between the ASD‐risk and Low‐risk recruitment groups (Chi‐square = 2.69, df = 3, *p* = .45).

Table 1 contains descriptive characteristics of the retained and not‐retained samples and Table 2 shows tests of predictors of study retention. Significant effects were found for household income, maternal education, and maternal age at child's birth, with higher levels of each variable associated with greater likelihood of retention. Travel distance was significantly different between the retained and not‐retained groups, with greater distance associated with lower likelihood of retention. This was not unexpected, as 16% of participants who were not retained moved over 400 miles from the study site after enrollment and chose to drop out of the study for this reason. In the retained group, only 2% lived over 400 miles from the study site. Outcome classification at exit also differed between the retained and not‐retained groups, with participants with ASD diagnoses more likely to be retained than those with typical development. More administrative contacts with families about enrollment and scheduling were significantly positively associated with retention.

**TABLE 2:** Predictors of study retention

Variable	Effect *χ* ^2^	Odds ratio (SE)	*p*‐value
Cohort	1.13		.57
Cohort 1/Cohort 2		0.99 (0.30)	.99
Cohort 1/Cohort 3		0.73 (0.25)	.35
Cohort 2/Cohort 3		0.73 (0.25)	.35
Recruitment group (ASD‐risk/Low‐risk)	0.89	0.77 (0.22)	.35
Sex of child (female/male)	0.73	0.80 (0.21)	.39
Minority status (White not Hispanic/Minority race ethnicity)	0.80	1.28 (0.35)	.37
Household income	8.27		.02
$100k+/$50k to $100k		1.47 (0.52)	.31
$100k+/$50k or less		2.70 (0.93)	.004
$50k to $100k/$50k or less		1.87 (0.66)	.08
Maternal education	9.54		.02
Graduate degree/College degree		1.79 (0.74)	.16
College degree/Some college		0.93 (0.34)	.85
Some college/High school or equivalent		2.98 (1.42)	.02
Maternal employment	4.45		.11
Full‐time/Part‐time		1.17 (0.51)	.72
Full‐time/Not working		1.94 (0.68)	.06
Part‐time/Not working		1.66 (0.62)	.18
Marital status (Married/Single)	0.09	1.19 (0.67)	.77
Maternal age at child birth (35 years/25 years)^a^	15.01	3.34 (1.06)	<.001
Travel distance (25 miles/150 miles)^a^	18.66	1.22 (0.07)	<.001
Number of children (1/3)^a^	0.87	0.31 (0.40)	.35
Intake general concerns (No/Yes)^a^	0.09	0.88 (0.38)	.77
Intake ASD concerns (No/Yes)^a^	0.47	1.30 (0.49)	.49
Outcome classification at exit	8.20		.02
ASD/Non‐TD		1.97 (1.32)	.32
ASD/TD		3.63 (2.23)	.04
Non‐TD/TD		1.85 (0.65)	.08
Proportion rescheduled visits (0/0.5)^a^	0.46	1.14 (0.22)	.50
Contacts re: Enrollment (4/1)^a^	5.14	1.35 (0.19)	.02
Contacts re: Scheduling per visit (4/1)^a^	56.68	36.7 (23.5)	<.001

^a^ Dichotomization of continuous variables done to illustrate magnitude of effects.

Retention rates did not differ by recruitment group (Chi‐square = 0.69, df = 1, *p* = .41), with roughly equivalent rates between the ASD‐risk (85.2%, CI = 80.9%–88.6%) and Low‐risk (87.9%, CI = 82.1%–92.0%) groups. Retention rates did not differ by cohort, with relatively equivalent retention rates (cohort 1: 83.9%, CI = 77.3%–88.8%, cohort 2: 83.9%, CI = 77.6%–88.7%), and cohort 3 (87.7%, CI = 81.1%–92.2%; Chi‐square = 1.13, df = 2, *p* = .57). There were also no differences between the retained and not‐retained groups in sex, race, ethnicity, age at enrollment, number of children in the family, maternal employment, marital status, or parent concerns about the child at enrollment.

Finally, we conducted a stepwise regression model, using all participant and family characteristics that differed significantly between retained and not‐retained families, to identify a set of uniquely significant predictors of study retention. Table 3 shows the parameter estimates and tests for all variables in the final stepwise model. Administrative contact variables were not included since they were not family‐level variables, but reflected lab operational procedures. Given that this analysis required all participants to have complete data on the relevant variables, a reduced sample of 371 participants with complete data was used. The stepwise regression model utilized a combination of forward and backward elimination, testing each variable at each step until a final set of significant predictors was identified. This procedure identified 4 variables that, together, provided the most parsimonious predictive model of study retention: Maternal education, maternal age at child's birth, travel distance, and outcome classification at exit. Together, these four variables explained 14.6% of the variance in retention, calculated as McFadden's pseudo R‐squared (McFadden, 1974), a relatively large effect size.

**TABLE 3:** Parameters of final stepwise model predicting study retention

Variable	Odds ratio	±95% CI	*t*‐value	*p*‐value
Intercept^a^	2.83	2.56–3.14	19.77	<.001
Maternal education				
High school or equivalent	0.80	0.71–0.91	−3.48	<.001
Some college	0.97	0.90–1.06	−0.65	.52
College degree	0.96	0.89–1.04	−0.98	.33
Maternal age at child birth	1.01	1.00–1.02	2.76	<.01
Travel distance	0.9996	0.9994–0.9998	−5.38	<.001
Outcome classification at exit				
Non‐TD	0.92	0.83–1.03	−1.48	.14
TD	0.91	0.83–0.99	−2.07	<.05

^a^ Reference categories for intercept are graduate level maternal education, maternal age at birth of 33.5 years, travel distance of 0 miles, and outcome of ASD.

## DISCUSSION

Reporting retention data (or its converse, attrition rates) is critical to determining the soundness of a study's conclusions (e.g., internal validity) and broader generalizability (e.g., external validity). Compromising internal or external validity can lead to overestimates of effects, biased conclusions, or overly expansive generalizations. The current study examined multiple factors potentially associated with retention in a large longitudinal study of younger siblings of children with ASD. We examined 14 child‐ and family‐level variables that were potentially associated with retention and found only 4 that contributed unique variance to predicting retention: maternal education and age at child birth, travel distance, and outcome classification (ASD, Non‐TD, TD) at the final visit. Not predictive of attrition were a number of variables critical to ensuring the validity of study results. Particularly important to external validity (or generalizability of study findings) were the lack of significant differences between the retained and not‐retained groups in sex, race, and ethnicity. Vital to internal validity (or soundness of the study's results) were the equivalence of the retained and not‐retained groups in familial ASD status (e.g., older affected sibling), age at enrollment, and parent concerns about the child at enrollment, any of which could have biased results had they been significantly different. There were also no differences between families who expressed initial interest in the study but did not enroll and those who did enroll. It is important to note that we did have missing data for some of the predictors of retention in our models and this may have attenuated our results if missing data was not completely random.

Travel distance was an expected predictor of attrition, based on both previous studies (Bradley et al., 2018; Ewing et al., 2022) and that over a third of those not retained cited a family relocation or travel issues as the primary reason for leaving the study prematurely. This suggests that longitudinal studies should be wary of enrolling families who live far from where data are collected and should consider including travel funds in their budgets to maximize retention for families who move out of the area after enrollment. Previous studies of varied samples have found that demographic variables like parental age and education level are also common predictors of both study enrollment (Bradshaw et al., 2020) and completion (Bradley et al., 2018; Ewing et al., 2022; Teixeira et al., 2021) and we replicated these findings in our sample as well. Lower household income, along with maternal age and education, were associated with greater attrition in our sample. This suggests that families with lower financial or educational resources may eventually determine that participation in a longitudinal research study is too burdensome to continue, unless additional support to maintain enrollment is provided.

The number of administrative contacts at both enrollment and later visits was also positively associated with retention in the study. This finding may seem counterintuitive, in that families who are non‐responsive and in danger of dropping out of a study would seem to require more outreach from study staff. However, families who agree to a visit then have multiple follow‐up contacts to agree upon a specific date and time and reminders of an upcoming visit, resulting in a significantly higher number of contacts than not‐retained families. Another factor driving the lower number of contacts in the not‐retained group was outdated contact information; although staff typically made multiple scheduling attempts, these would be aborted if phone numbers or email addresses were no longer in service. A higher number of administrative contacts may also reflect greater responsivity to scheduling negotiations, greater willingness to reschedule an appointment, and greater likelihood of updating contact information if changed.

One finding that does have implications for internal validity is the significantly higher rate of retention of children who were diagnosed with ASD during the course of the study than of children who were found to be typically developing. While retention of participants with typical development was still high (83%), it was significantly lower than retention of participants with ASD (95%). Greater loss of typically developing participants could, in particular, inflate estimates of recurrence risk. A large international multi‐site study conducted by the Baby Siblings Research Consortium reported an 18.7% recurrence rate of ASD in families with an affected older sibling (Ozonoff et al., 2011). That study did not report retention data, since it was not collected systematically by the many sites contributing to the analyses, so it is unknown how attrition may have affected recurrence rate. It will be critical for future studies to explicitly address this issue in order to calculate the most accurate and unbiased recurrence rates for families who wish to have this information. If future studies, like the current study, report higher retention of participants diagnosed with ASD, this may suggest that the sibling recurrence is lower than the 18.7% reported previously (Ozonoff et al., 2011).

Many publications describe engagement and tracking methods that can be used to maximize retention (e.g., Abshire et al., 2017; Cotter et al., 2002, 2005; Scott, 2004). Teague et al. (2018), in their meta‐analysis of retention in longitudinal cohorts, identified 95 innovative strategies used across research projects to improve study completion. These strategies fell into four categories: scheduling and reminders, community building, tracking methods, and barrier reduction, all of which we utilized to improve retention across the 3 years of participation required by our longitudinal study. We had a detailed protocol and tracking database for visit scheduling and reminders. We contacted families between visits with occasional lab newsletters detailing new findings and invitations to recreational and community events. We provided reports for all participants (including typically developing children) with information about developmental functioning and referrals to services when needed, which were highly valued by parents. We had institutional approval to collect next‐of‐kin locator information and use public records tracking services when we could not reach a family. We offered travel funds, from reimbursement for gas, to airline tickets and hotel rooms for families who had moved but were willing to travel for study visits if costs could be covered. These strategies contributed to a high retention rate of 85% from enrollment near birth through the third birthday. They may also have minimized the potential impact on retention of demographic factors (race, ethnicity) or family stressors (number of children, maternal employment, marital status), none of which were associated with study completion in this sample. This study found relatively few threats to internal and external validity due to selective retention, suggesting that findings from this lab, and others using similar longitudinal methods with similar samples and similar research questions, are producing largely accurate results that are generalizable to the broader community of families with a child with ASD. It will be critical, however, for future infant sibling studies to track, report, and account analytically for attrition to fully understand the potential effects of selective retention on study results, particularly of sibling recurrence rates.

## AUTHOR CONTRIBUTIONS

**Sally Ozonoff**: Conceptualization; Funding acquisition; Investigation; Methodology; Project administration; Resources; Supervision; Validation; Writing – original draft. **Monique M. Hill**: Conceptualization; Data curation; Investigation; Project administration; Writing – review & editing. **Alesha Hill**: Conceptualization; Data curation; Investigation; Methodology; Project administration; Writing – review & editing. **Kevin Ashley**: Data curation; Formal analysis; Visualization; Writing – review & editing. **Gregory S. Young**: Conceptualization; Data curation; Formal analysis; Investigation; Methodology; Software; Validation; Visualization; Writing – review & editing.

## CONFLICTS OF INTEREST

Sally Ozonoff has received research grant funding from the National Institutes of Health and Autism Speaks; travel reimbursement and honoraria for editorial activities from Autism Speaks, Autism Science Foundation, and Wiley; and book royalties from Guilford Press and American Psychiatric Press, Inc. Gregory S. Young has received research grant funding from the Autism Science Foundation. The remaining authors have declared that they have no competing or potential conflicts of interest.

## ETHICAL CONSIDERATIONS

All procedures were approved by the University of California Davis Institutional Review Board.

## Data Availability

The data that support the findings of this study are available from the corresponding author upon reasonable request.
